# Early Phase I Cardiac Rehabilitation Integrated with Multidisciplinary Post-Acute Care in Decompensated Heart Failure: Insights from Serial Cardiopulmonary Exercise Testing

**DOI:** 10.3390/medicina61061080

**Published:** 2025-06-12

**Authors:** Ruei-Sian Ding, Ko-Long Lin, Wen-Hwa Wang, Ming-Hsuan Huang, I-Hsiu Liou

**Affiliations:** 1 Department of Physical Medicine and Rehabilitation, Kaohsiung Veterans General Hospital, Kaohsiung 813, Taiwan; 2 Department of Physical Medicine and Rehabilitation, Kaohsiung Medical University Hospital, Kaohsiung Medical University, Kaohsiung 807, Taiwan; klin967@gmail.com; 3 Department of Physical Medicine and Rehabilitation, School of Medicine, College of Medicine, Kaohsiung Medical University, Kaohsiung 807, Taiwan; 4 Cardiovascular Center, Kaohsiung Veterans General Hospital, Kaohsiung 813, Taiwan; 5 Department of Physical Therapy, School of Medical and Health Science, Fooyin University, Kaohsiung 831, Taiwan

**Keywords:** acute decompensated heart failure, cardiac rehabilitation, post-acute care, cardiopulmonary exercise testing, peak oxygen uptake, multidisciplinary team, heart failure

## Abstract

*Background and Objectives:* Acute decompensated heart failure (ADHF) leads to significant impairments in exercise capacity and functional outcomes. This study aimed to evaluate the feasibility and effectiveness of integrating early phase I cardiac rehabilitation with a multidisciplinary heart failure post-acute care (HF-PAC) program to improve functional capacity in patients hospitalized for ADHF, assessed by serial cardiopulmonary exercise testing (CPET). *Materials and Methods:* We conducted a prospective cohort study at a medical center in Taiwan. Patients hospitalized for ADHF between February 2017 and March 2023 who completed inpatient and six-month follow-up CPET were enrolled. The rehabilitation protocol included supervised aerobic and resistance training during hospitalization, followed by outpatient multidisciplinary care. The primary outcome was the change in peak oxygen uptake (peak VO_2_) over six months. *Results:* A total of 90 patients were included (74.4% male, mean age 58.4 ± 14.7 years). Peak VO_2_ significantly improved from 11.57 ± 3.33 to 13.99 ± 4.2 mL/kg/min (*p* < 0.001). Significant improvements were also observed in 6 min walk distance, anaerobic threshold, heart rate recovery, oxygen uptake efficiency slope, and left ventricular ejection fraction. *Conclusions:* Early integration of phase I cardiac rehabilitation with multidisciplinary HF-PAC is feasible and enhances exercise capacity in patients with ADHF. Serial CPET provides an objective evaluation of functional recovery and may guide rehabilitation strategies in this high-risk population.

## 1. Introduction

Heart failure (HF) affects approximately 1.9% to 2.6% of the US adult population [1]. In Taiwan, the estimated prevalence is notably higher, at around 6% [2], a discrepancy that may be attributed to population aging and the National Health Insurance system, which facilitates timely access to care [3,4]. These epidemiological trends underscore the need for comprehensive, long-term rehabilitation strategies to address the functional decline and high risk of rehospitalization in this population.

Cardiac rehabilitation (CR) confers multiple cardiopulmonary benefits, including enhanced aerobic capacity, improved peripheral vascular and endothelial function, reduction in depressive symptoms, and facilitation of left ventricular diastolic function, which is associated with improved clinical outcomes [5]. There is growing consensus that structured, multidisciplinary care significantly enhances both quality of life and functional capacity in patients with chronic HF [6]. Notably, early-phase exercise interventions have also demonstrated feasibility and safety in patients with acute decompensated heart failure (ADHF), contributing to reductions in all-cause rehospitalization rates and improvements in 6 min walk distance (6MWD) [7,8,9].

The Heart Failure Post-Acute Care (HF-PAC) program is a nationwide initiative launched by Taiwan’s National Health Insurance Administration, designed to deliver patient-centered care through multidisciplinary team (MDT) collaboration. Patients with ADHF are enrolled during hospitalization and receive coordinated care that includes individualized education, self-care training, and discharge planning for both patients and caregivers [10]. The MDT typically comprises cardiologists, physiatrists, nurses, case managers, rehabilitation therapists, dietitians, pharmacists, social workers, and psychologists. Following discharge, patients continue rehabilitation through structured cardiopulmonary programs, regular outpatient follow-up, and remote support via telephone. This integrated care model has been shown to improve medication adherence, enhance self-management, support reintegration into the community, and significantly reduce the rate of hospital readmission.

The cardiopulmonary exercise test (CPET) remains the gold standard for assessing functional capacity and prognostic status in HF patients [11]. However, individuals with ADHF frequently present with severe physical deconditioning and frailty, often rendering them unable to complete maximal exertion protocols. As such, the six-minute walking test (6MWT) is commonly employed as a submaximal, safer alternative during the early stages of recovery [12]. Although widely used, the role and feasibility of CPET across different phases of rehabilitation in this population remain underexplored.

This study aimed to evaluate the feasibility and clinical utility of CPET during the transition from inpatient (phase I) to outpatient CR in patients hospitalized for acute HF. We further sought to assess changes in functional capacity using CPET as a standardized outcome measure following a six-month multidisciplinary rehabilitation program.

## 2. Materials and Methods

### 2.1. Study Population and Intervention

This prospective cohort study enrolled patients hospitalized for heart failure at a single tertiary medical center in Taiwan between February 2017 and March 2023. Eligible participants were aged >18 years and had either newly diagnosed or acutely decompensated heart failure. All included patients completed at least two CPETs, one during hospitalization and another at 6-month follow-up.

Exclusion criteria included: (1) excessive physical debilitation precluding CPET completion; (2) cognitive impairment or neuromuscular disease with poor rehabilitation potential; (3) bedridden status for more than three months; (4) loss to follow-up during outpatient care; and (5) dependence on mechanical ventilation or severe pulmonary disease requiring long-term oxygen therapy.

During hospitalization, patients were managed primarily by cardiologists who oversaw heart failure-specific pharmacological therapy. Once patients achieved clinical stability—defined by the absence of acute complications and hemodynamic instability—a rehabilitation consultation was initiated for phase I CR.

The rehabilitation protocol, adapted from the American College of Sports Medicine (ACSM) guidelines and modified for institutional use at Kaohsiung Veterans General Hospital, included resistance training, endurance training, sitting and transfer training, and ambulation as tolerated. Exercise intensity was titrated to achieve a heart rate increase of approximately 20 beats per minute above baseline. All physical therapists involved in the program were certified in cardiopulmonary rehabilitation and had at least three years of relevant clinical experience.

Prior to discharge, patients underwent baseline CPET and a 6MWT. A follow-up assessment, including both tests, was performed six months post-discharge. This study was approved by the Institutional Review Board of Kaohsiung Veterans General Hospital (IRB VGHKS17-CT11-11). Written informed consent was obtained from all participants prior to enrollment.

### 2.2. Exercise Testing

CPET was conducted using the MetaLyzer 3B system (Cortex Biophysik GmbH, Leipzig, Germany), which includes a leg ergometer, breath-by-breath gas analyzer, and 12-lead electrocardiography for continuous monitoring. Each test employed a ramp protocol with an incremental workload of 10 watts per minute and was supervised by a board-certified physiatrist with over 10 years of experience.

During CPET, the following parameters were directly measured: oxygen consumption (VO_2_), carbon dioxide production (VCO_2_), minute ventilation (VE), and respiratory rate. Derived indices included the respiratory exchange ratio (RER), ventilatory efficiency (VE–VCO_2_ slope), anaerobic threshold (AT), and oxygen uptake efficiency slope (OUES). AT was identified by a nonlinear increase in the VCO_2_–VO_2_ slope. The OUES was calculated using the linear regression formula VO_2_ = a·log(VE) + b, where the coefficient a represents OUES. Exercise testing was terminated when participants reached their self-reported maximal exertion or at the discretion of the supervising physician based on safety concerns.

### 2.3. Statistical Analysis

All statistical analyses were performed using IBM SPSS Statistics version 20.0 (IBM Corp., Armonk, NY, USA). Continuous variables are expressed as means ± standard deviation, while categorical variables are presented as frequencies and percentages. Paired *t*-tests were used to compare pre- and post-rehabilitation values of exercise performance. Pearson correlation coefficients were calculated to explore associations among 6MWT distance, peak VO_2_, and other exercise-related parameters. A two-tailed *p*-value of <0.05 was considered statistically significant.

## 3. Results

A total of 90 patients with ADHF were enrolled—67 males and 23 females. The mean age was 58.38 ± 14.7 years, and the mean BMI was 25.46 ± 6.72 kg/m^2^. The baseline demographics, heart failure etiology, and comorbidities are summarized in Table 1. The most common primary etiology of ADHF was coronary artery disease (46.7%), followed by dilated cardiomyopathy (14.4%) and mitral regurgitation (14.4%). The most frequent comorbidities were hypertension, diabetes mellitus, and dyslipidemia.

Table 2 presents the cardiopulmonary exercise capacity and echocardiographic parameters measured at phase I CR and at a six-month follow-up. During hospitalization, the average left ventricular ejection fraction (LVEF) was 31.38 ± 8.31%. Peak oxygen uptake (peak VO_2_) significantly improved from 11.57 ± 3.33 mL/kg/min at baseline to 13.99 ± 4.2 mL/kg/min at follow-up (*p* < 0.001). There were no statistically significant differences in RER or VO_2_–WR slope between the two assessments. However, significant improvements were observed in the following parameters: peak VE (*p* = 0.001), peak heart rate (*p* < 0.001), 6MWD (*p* = 0.01), heart rate recovery (HRR) (*p* = 0.014), anaerobic threshold oxygen uptake (ATVO_2_) (*p* < 0.001), VE–VCO_2_ slope (*p* = 0.011), OUES (*p* = 0.001), and LVEF (*p* < 0.001). No adverse events occurred during CPET or supervised rehabilitation sessions.

Table 3 shows the Pearson correlation coefficients for the changes observed in each parameter. Changes in 6MWT demonstrated a significant positive correlation with changes in both peak RER (r = 0.314, *p* = 0.003) and peak VO_2_ (r = 0.346, *p* = 0.001). No other correlations reached statistical significance in this comparison.

As shown in Table 4, improvements in peak VO_2_ were significantly correlated with changes in ATVO_2_ (r = 0.677, *p* < 0.01), HRR (r = 0.259, *p* = 0.014), VE–VCO_2_ slope (r = −0.249, *p* = 0.019), OUES (r = 0.461, *p* < 0.01), LVEF (r = 0.677, *p* < 0.01), and 6MWD (r = 0.346, *p* = 0.001). Although several associations reached statistical significance, their effect sizes were classified as weak to moderate (r ≤ 0.35 = weak; 0.36–0.67 = moderate). For example, the change in 6MWD showed only a weak correlation with the change in peak VO_2_ (r = 0.346) and with the change in peak RER (r = 0.314) (Table 3).

## 4. Discussion

Previous studies have demonstrated that patients with ADHF exhibit impaired physiological parameters, such as reduced oxygen uptake and cardiac output [15], both of which are associated with poor prognosis and increased rates of hospital readmission. Early cardiopulmonary rehabilitation has been shown to improve these outcomes. In our study, participation in a phase I cardiopulmonary rehabilitation program integrated within a multidisciplinary HF-PAC program significantly enhanced exercise tolerance in ADHF patients quantified by serial CPET assessments.

Compared with conventional care, HF-PAC with MDT-based interventions provide more comprehensive support—including physiological, psychological, and nutritional components—which facilitate more effective recovery of cardiopulmonary function. This is consistent with previous findings indicating that MDT-based care for ADHF can reduce hospital readmissions and mortality while improving exercise performance and activities of daily living [16,17]. These findings collectively support the early initiation of MDT-led interventions during hospitalization, followed by continued post-discharge care, to maximize the benefits of cardiopulmonary rehabilitation in this high-risk population.

Following acute heart failure, patients frequently experience declines in muscular strength, endurance, and overall functional capacity, which are closely associated with increased rates of hospital readmission, reduced ADL performance, and higher mortality risk [7]. Previous studies have established that early rehabilitation initiated during hospitalization is both safe and effective in improving functional outcomes and reducing readmission rates in patients with ADHF. To evaluate the efficacy of interventions, a variety of multiparametric clinical tools are routinely used in practice. Among them, the 6MWT and independent gait performance are commonly applied and have shown utility in tracking changes before and after rehabilitation [7,18,19]. Motoki et al. [20] further suggested that inpatient cardiopulmonary rehabilitation should be initiated as soon as the patient’s hemodynamic status stabilizes, with the Barthel index serving as a practical and reliable measure of functional recovery. In addition to functional scales, several laboratory biomarkers—such as plasma volume status, brain natriuretic peptide (BNP), blood urea nitrogen, and arterial blood gas values—have been identified as useful predictors of long-term mortality in ADHF populations [21,22]. Furthermore, Doppler ultrasonography to assess intrarenal blood flow during hospitalization has also been proposed as a potential prognostic tool for adverse outcomes and mortality [23]. As is well recognized, CPET is the gold standard for evaluating exercise capacity in patients with heart failure [11]. However, its availability remains limited in many clinical settings, and in ADHF populations, achieving maximal effort during CPET is often challenging due to safety concerns and physiological limitations. Our study further supports the feasibility and safety of conducting CPET in the acute setting and highlights its value in providing objective and quantifiable parameters to assess pre- and post-intervention changes.

Beyond its role as an objective measure of exercise capacity, CPET provides valuable prognostic and longitudinal indicators for patients with heart failure. Among these, peak VO_2_ remains one of the most widely used metrics. In patients with chronic systolic HF, peak VO_2_ < 10.0 mL/kg/min has been associated with poor prognosis [24], while a commonly used cutoff value of 14 mL/kg/min has been shown to predict 1-year mortality [25,26]. For patients with congestive heart failure due to ischemic or dilated cardiomyopathy, predicted VO_2_ max ≤ 50% of age-adjusted normal values is also considered an important prognostic marker [27]. In our study, 73 (81.1%) patients in group had peak VO_2_ below 14 mL/kg/min at baseline. After six months of cardiopulmonary rehabilitation, this proportion had improved significantly, with 50% of patients exceeding the 14 mL/kg/min threshold, highlighting the potential of structured rehabilitation in improving functional prognosis in the ADHF population. However, achieving maximum exercise effort is often limited in acute patients due to common symptoms such as exercise intolerance. RER ≥ 1.1 is typically used as a criterion for peak effort [24]. In our study, only 45 patients in the group, about 51.1%, met this threshold. While the proportion did not differ significantly before and after rehabilitation, prior studies have shown that nearly half of HF patients failed to achieve RER ≥ 1.1, even under stable conditions [28,29]. In our cohort, average RER during both tests ranged from 1.08 to 1.09. Although these values did not reach the conventional maximal effort criterion of ≥1.1, they are considered acceptable in patients with ADHF, given their limited exercise tolerance. Contemporary heart-failure cohorts likewise retain tests with peak RER > 1.05 and still confirm the prognostic validity of peak VO_2_ and ventilatory-efficiency indices [11,30,31]. This suggests that the CPETs were conducted with sufficient intensity to provide reliable physiological data. In such contexts, submaximal functional capacity indicators become particularly valuable and clinically relevant for assessing treatment response.

Beyond statistical significance, the observed functional gains also met accepted minimal clinically important differences (MCIDs). While the MCID for peak VO_2_ on CPET has not been formally validated, a commonly cited threshold of ≈1 mL/kg/min or a 6% relative rise, particularly in patients with substantially reduced baseline exercise tolerance [32], is widely referenced in contemporary regulatory guidance. Our cohort improved by 2.42 mL/kg/min (+20.9%), thus exceeding this benchmark more than twofold. Similarly, a previous study in chronic heart failure identified a 32 m improvement in 6MWD as the MCID for this population [33]. We recorded a mean gain of 65 m, more than double the upper limit of that clinically meaningful range. These considerations reinforce that the functional enhancements we report are not only statistically robust but also perceptible and valuable to patients.

Among submaximal exercise parameters, the VE–VCO_2_ slope is frequently used in heart failure populations, with a value ≥ 45 indicating poor prognosis (normal < 30). Additional indices—such as AT, HRR, 6MWT, and OUES—are also recognized as valuable tools for assessing exercise tolerance and guiding prognostic evaluation [24]. In our study, the observed improvements in these submaximal parameters further support the clinical value of cardiopulmonary rehabilitation combined with multidisciplinary intervention. Importantly, these markers may be used individually or in combination to enhance risk stratification. For example, Gitt et al. reported that in a cohort of 223 patients with chronic heart failure, the combination of O_2_ at AT < 11 mL/kg/min and VE–VCO_2_ slope > 34 significantly predicted 6-month mortality [34]. Given that many older patients may be unable to complete maximal exercise testing or reach peak effort due to age-related limitations [35], submaximal indices offer a feasible and clinically meaningful alternative in this population. They provide prognostic insight even in the absence of maximal CPET effort, and should be considered essential tools in the functional evaluation of HF patients.

In patients with advanced heart failure, previous studies—including that by Lipkin et al. [36]—have reported a significant correlation between 6MWT distance and peak VO_2_. In our study, we observed a similar positive correlation between 6MWT and peak VO_2_ in patients with ADHF, both in single-point measurements and in the degree of improvement after six months of cardiopulmonary rehabilitation (r = 0.346, *p* < 0.05). These findings further validate the 6MWT as a reliable surrogate marker for cardiopulmonary exercise capacity in this patient population. Moreover, the observed trend of parallel improvement between 6MWT and peak VO_2_ supports the use of 6MWT in clinical settings as a practical tool for monitoring changes in functional capacity. Nevertheless, our study also confirmed that CPET is both safe and feasible in ADHF patients when performed under appropriate medical supervision. CPET provides a more comprehensive physiological profile, offering detailed insights into cardiopulmonary adaptation and prognostic risk. While the 6MWT is frequently used due to its simplicity and accessibility, our findings emphasize the complementary role of CPET in this population. Together, these two tests serve as valuable reference tools for evaluating pre- and post-intervention changes in cardiopulmonary function, enhancing the precision of functional assessment in ADHF care.

In our study, a significant positive correlation was observed between LVEF and peak VO_2_ following six months of cardiopulmonary rehabilitation (r = 0.677, *p* < 0.05). In contrast, no significant association was found between LVEF and 6MWT distance (r = 0.169, *p* = 0.12). This finding supports the utility of peak VO_2_ as a core indicator of cardiopulmonary exercise capacity, reflecting improvements in cardiac function more sensitively than submaximal measures. While the 6MWT is correlated with peak VO_2_ and may serve as a practical alternative in submaximal testing, its results are influenced by a variety of factors, such as lower-extremity strength, gait stability, and daily functional status. These confounding elements may account for the weaker correlation with echocardiographic indices such as LVEF. Moreover, LVEF itself is subject to variability based on image quality, measurement technique, and operator expertise [37], which may limit its reliability in longitudinal assessments. Therefore, for patients with ADHF, CPET remains the preferred tool for evaluating exercise capacity, particularly in assessing cardiopulmonary adaptation and long-term prognosis. Nevertheless, in resource-limited settings or in patients who are unable to tolerate CPET, echocardiography remains a valuable adjunct for monitoring cardiac function and guiding therapeutic decisions.

In patients with ADHF, early CPET has been shown to play a critical role in both risk stratification and therapeutic planning. Previous research has identified the OUES as a powerful prognostic indicator, with one study reporting that patients with an OUES < 1.25 at discharge had a 5.4-fold increased risk of experiencing major adverse cardiovascular events within one year [38]. Beyond “classic” ischemic or dilated etiologies, CPET has also proved valuable in infiltrative cardiomyopathies. Cardiac amyloidosis—particularly transthyretin- and light-chain–related forms—often presents clinically as HFpEF and may progress to acute decompensation. Recent evidence synthesized by Pugliatti et al. demonstrated that markedly reduced peak VO_2_ and steep VE–VCO_2_ slopes on CPET independently predict mortality and guide decisions on disease-modifying therapy, underscoring the versatility of CPET across diverse heart-failure phenotypes [39]. Our findings further support the role of CPET as a central tool for functional assessment in ADHF, providing precise physiological data to guide individualized rehabilitation strategies. Notably, our study found a significant correlation between improvements in peak VO_2_ and LVEF, whereas no such relationship was observed with the 6MWT. This underscores the superiority of CPET in detecting changes in cardiopulmonary adaptation over time. Based on these findings, we recommend that CPET be performed during the phase I rehabilitation period to obtain a comprehensive baseline assessment of exercise capacity, which can serve as the foundation for designing a tailored phase II rehabilitation program. In clinical settings where CPET is not readily available due to equipment or staffing limitations, a combination of echocardiography and the 6MWT may offer a feasible alternative for monitoring cardiac function and functional recovery. While not as detailed as CPET, this combined approach can still provide valuable insight into patient status and guide subsequent intervention planning.

Several limitations should be acknowledged when interpreting the findings of this study. First, this was a single-arm prospective cohort without a standard-care control group. Consequently, causal inference is limited and the observed improvements may partly reflect natural recovery or co-interventions. Second, this was a single-center study conducted at a veterans’ medical hospital, and 74% of participants were male. Such a sampling frame may introduce selection bias: veterans are predominantly male, may differ socio-demographically from the general population, and often present with comorbidity profiles. The relatively small sample further limits the generalizability of our findings. Third, the study primarily relied on functional outcome measures, such as CPET and the 6MWT, and did not serially collect BNP or other biomarkers, as these assays were not incorporated into the routine clinical workflow during the study period. The inclusion of such biomarkers might have offered additional insight into treatment response. Moreover, because most correlations were weak or moderate, their independent explanatory power is limited and should therefore be interpreted alongside other clinical information. Fourth, outcome assessors were not blinded, so measurement bias cannot be entirely excluded. Fifth, follow-up was restricted to six months, so the durability of the observed benefits beyond this period remains uncertain. Finally, repeated functional tests may introduce a learning effect. Controlled studies have shown that walking-naïve patients can improve their 6MWD distance simply through familiarization [40]. Although prior work suggests minimal familiarization for CPET in heart-failure populations [25], a modest training or motivation effect cannot be completely excluded.

Future investigations should therefore incorporate multicenter enrolment, larger and more gender-balanced samples, and randomized or matched control groups to strengthen causal inference and generalizability. In addition, serial biomarker assessment, blinded outcome evaluation, and long-term follow-up are needed to further validate and extend these findings.

## 5. Conclusions

In summary, this study demonstrates that early cardiopulmonary rehabilitation combined with a multidisciplinary HF-PAC program can significantly improve exercise capacity in patients with ADHF, as evidenced by increased peak VO_2_ and its correlation with 6MWT improvement. While submaximal tests like the 6MWT offer practical alternatives, CPET remains the most comprehensive tool for assessing functional recovery and guiding individualized rehabilitation. We recommend incorporating CPET during phase I rehabilitation to establish a robust baseline and inform phase II planning. In settings with limited resources, the 6MWT and echocardiography may serve as feasible substitutes, though with acknowledged limitations.

## Figures and Tables

**Table 1:** Baseline characteristics of all enrolled patients.

All Patients (*n* = 90)		
	*n* (%)	Mean ± SD
Age (year)		58.38 ± 14.70
Gender		
Male	67 (74.4)	
Female	23 (25.5)	
Height (cm)		165.08 ± 8.79
Weight (kg)		70.29 ± 23.21
BMI (kg/m^2^)		25.46 ± 6.72
NYHA classification		
I	1 (1.1)	
II	30 (33.3)	
III	54 (60.0)	
IV	5 (5.5)	
ADHF etiology		
DCM	13 (14.4)	
MR	13 (14.4)	
CAD ^†^	42 (46.7)	
Myocarditis	4 (4.4)	
Thrombus	2 (2.2)	
Other ^‡^	2 (2.2)	
Unknown	14 (15.6)	
Comorbidities		
Hypertension	56 (62.2)	
COPD	6 (6.6)	
DM	35 (38.8)	
PAOD	2 (2.2)	
ESRD	3 (3.3)	
Dyslipidemia	45 (50)	

† CAD includes ischemic heart disease and ischemic cardiomyopathy. ‡ “Other” includes single cases of restrictive cardiomyopathy (RCM) and peripartum cardiomyopathy (PPCM). BMI = body mass index; NYHA = New York Heart Association; ADHF = acute decompensated heart failure; DCM = dilated cardiomyopathy; MR = mitral regurgitation; CAD = coronary artery disease; COPD = chronic obstructive pulmonary disease; DM = diabetes mellitus; PAOD = peripheral arterial occlusion disease; ESRD = end-stage renal disease.

**Table 2:** Changes in CPET parameters between baseline and six-month follow-up.

	Number	Baseline	After Six Months	Mean Difference (95% CI)	*p*-Value
	n	Mean ± SD	Mean ± SD		
Peak VO_2_ (mL/min/kg)	90	11.57 ± 3.33	13.99 ± 4.2	+2.42 (+1.75 to +3.07)	*p* < 0.001
Peak VE (L/min)	90	34.06 ± 11.45	37.88 ± 12.57	+3.82 (+1.63 to +6.01)	*p* = 0.001
Peak HR	90	107.8 ± 21.26	115.81 ± 22.3	+8.01 (+3.73 to +12.28)	*p* < 0.001
Peak RER	90	1.08 ± 0.09	1.09 ± 0.08	+0.01 (−0.01 to +0.03)	*p* = 0.298
6MWD (m)	87	299.96 ± 107.56	365.44 ± 103.26	+65.48 (+47.94 to +83.01)	*p* = 0.010
HRR	89	11.6 ± 8.49	14.74 ± 9.95	+3.14 (+0.64 to +5.62)	*p* = 0.014
ATVO_2_ (mL/min/kg)	89	8.17 ± 2.38	9.98 ± 2.97	+1.81 (+1.30 to +2.30)	*p* < 0.001
VE–VCO_2_ slope	89	37.78 ± 11	34 ± 14.67	−3.78 (−0.88 to −6.66)	*p* = 0.011
OUES	89	1.14 ± 0.44	1.39 ± 0.61	+0.25 (+0.14 to +0.36)	*p* = 0.001
LVEF (%)	90	31.38 ± 8.31	42.67 ±11.09	+11.29 (+9.02 to +13.54)	*p* < 0.001
VO_2_–WR slope	89	7.44 ± 2.9	7.79 ± 3.08	+0.35 (−0.38 to +1.07)	*p* = 0.349
Maximal workload (W)	90	48.44 ± 20.24	63.63 ± 29.01	+15.19 (+10.62 to +19.75)	*p* < 0.001

SD = standard deviation; VO_2_ = oxygen uptake; VE = minute ventilation; HR = heart rate; RER = respiratory exchange ratio; 6MWD = six-minute walking distance; HRR = heart rate recovery; AT = anaerobic threshold; VCO_2_ = volume of exhaled carbon dioxide; OUES = oxygen uptake efficiency slope; LVEF = left ventricular ejection fraction; WR = work rate; CI = confidence interval.

**Table 3:** Association between 6MWD improvement and cardiopulmonary parameters.

Variable	n	r	*p*-Value
ΔATVO_2_ (mL/min/kg)	86	0.169	0.120
ΔPeak RER	87	0.314	0.003 *
ΔHRR	86	0.020	0.855
ΔVE–VCO_2_ slope	86	−0.197	0.069
ΔOUES	86	0.166	0.127
ΔLVEF (%)	86	0.169	0.120
ΔPeak VO_2_ (mL/kg/min)	87	0.346	0.001 *

All values are Pearson correlation coefficients (r). Effect-size thresholds: r ≤ 0.35 = weak; 0.36–0.67 = moderate; ≥0.68 = strong [13,14]. * *p* < 0.05; Δ = change from baseline to 6 months; AT = anaerobic threshold; HRR = heart rate recovery; VO_2_ = oxygen uptake; RER = respiratory exchange ratio; VE = minute ventilation; VCO_2_ = volume of exhaled carbon dioxide; OUES = oxygen uptake efficiency slope; LVEF = left ventricular ejection fraction.

**Table 4:** Association between peak VO_2_ improvement and cardiopulmonary parameters.

Variable	n	r	*p*-Value
ΔATVO_2_ (mL/min/kg)	89	0.677	<0.01 *
ΔPeak RER	90	0.180	0.090
ΔHRR	89	0.259	0.014 *
ΔVE–VCO_2_ slope	89	−0.249	0.019 *
ΔOUES	89	0.461	<0.01 *
ΔLVEF (%)	89	0.677	<0.01 *
Δ6MWT (m)	87	0.346	0.001 *

All values are Pearson correlation coefficients (r). Effect-size thresholds: r ≤ 0.35 = weak; 0.36–0.67 = moderate; ≥0.68 = strong [13,14]. * *p* < 0.05; Δ = difference between follow-up and baseline values; AT = anaerobic threshold; HRR = heart rate recovery; VO_2_ = oxygen uptake; RER = respiratory exchange ratio; VE = minute ventilation; VCO_2_ = volume of exhaled carbon dioxide; OUES = oxygen uptake efficiency slope; LVEF = left ventricular ejection fraction; 6MWT = six-minute walking test.

## Data Availability

The original contributions presented in this study are included in the article. Further inquiries can be directed to the corresponding author.

## Abbreviations

The following abbreviations are used in this manuscript:

6MWD	6 min walk distance
6MWT	6 min walk test
ACSM	American College of Sports Medicine
ADHF	acute decompensated heart failure
AT	anaerobic threshold
BNP	brain natriuretic peptide
CAD	coronary artery disease
COPD	chronic obstructive pulmonary disease
CPET	cardiopulmonary exercise testing
CR	cardiac rehabilitation
DCM	dilated cardiomyopathy
DM	diabetes mellitus
ESRD	end-stage renal disease
HF	heart failure
HF-PAC	heart failure post-acute care
HRR	heart rate recovery
LVEF	left ventricular ejection fraction
MDT	multidisciplinary team
MR	mitral regurgitation
NYHA	New York Heart Association
OUES	oxygen uptake efficiency slope
PAOD	peripheral arterial occlusion disease
Peak VO_2_	peak oxygen uptake
RER	respiratory exchange ratio
SD	standard deviation
VCO_2_	carbon dioxide production
VE	minute ventilation
VO_2_	oxygen consumption
WR	work rate
